# A multi-center interventional study to assess pharmacokinetics, effectiveness, and tolerability of prolonged-release tacrolimus after pediatric kidney transplantation: study protocol for a prospective, open-label, randomized, two-phase, two-sequence, single dose, crossover, phase III b trial

**DOI:** 10.3389/fneph.2024.1331510

**Published:** 2024-02-20

**Authors:** Sinem Karaterzi, Burkhard Tönshoff, Thurid Ahlenstiel-Grunow, Maral Baghai, Bodo Beck, Anja Büscher, Lisa Eifler, Thomas Giese, Susanne Lezius, Carsten Müller, Jun Oh, Antonia Zapf, Lutz T. Weber, Lars Pape

**Affiliations:** ^1^ Department of Pediatrics II, University Hospital of Essen, Essen, Germany; ^2^ Department of Pediatrics I, University Children’s Hospital Heidelberg, Heidelberg, Germany; ^3^ Structural and Computational Biology Unit, European Molecular Biology Laboratory, Heidelberg, Germany; ^4^ Institute of Human Genetics, University Hospital of Cologne, Cologne, Germany; ^5^ Children’s and Adolescents’ Hospital, Pediatric Nephrology, University Hospital of Cologne, Cologne, Germany; ^6^ Department of Immunology, University Hospital Heidelberg, Heidelberg, Germany; ^7^ Institute of Medical Biometry and Epidemiology, University Hospital Eppendorf, Hamburg, Germany; ^8^ Pharmacology at the Laboratory Diagnostics Centre, Faculty of Medicine and University Hospital Cologne, University of Cologne, Cologne, Germany; ^9^ Department of Pediatric Nephrology and Gastroenterology, University Hospital Eppendorf, Hamburg, Germany

**Keywords:** tacrolimus, Prograf^®^, Envarsus^®^, pharmacokinetics, pharmacodynamics, pharmacogenetics, adherence, kidney transplantation

## Abstract

**Background:**

Tacrolimus, a calcineurin inhibitor (CNI), is currently the first-line immunosuppressive agent in kidney transplantation. The therapeutic index of tacrolimus is narrow due to due to the substantial impact of minor variations in drug concentration or exposure on clinical outcomes (i.e., nephrotoxicity), and it has a highly variable intra- and inter-individual bioavailability. Non-adherence to immunosuppressants is associated with rejection after kidney transplantation, which is the main cause of long-term graft loss. Once-daily formulations have been shown to significantly improve adherence compared to twice-daily dosing. Envarsus^®^, the once-daily prolonged-release formulation of tacrolimus, offers the same therapeutic efficacy as the conventional twice-daily immediate-release tacrolimus formulation (Prograf^®^) with improved bioavailability, a more consistent pharmacokinetic profile, and a reduced peak to trough, which may reduce CNI-related toxicity. Envarsus^®^ has been approved as an immunosuppressive therapy in adults following kidney or liver transplantation but has not yet been approved in children. The objective of this study is to evaluate the pharmacokinetic profile, efficacy, and tolerability of Envarsus^®^ in children and adolescents aged ≥ 8 and ≤ 18 years to assess its potential role as an additional option for immunosuppressive therapy in children after kidney transplantation.

**Methods/design:**

The study is designed as a randomized, prospective crossover trial. Each patient undergoes two treatment sequences: sequence 1 includes 4 weeks of Envarsus^®^ and sequence 2 includes 4 weeks of Prograf^®^. Patients are randomized to either group A (sequence 1, followed by sequence 2) or group B (sequence 2, followed by sequence 1). The primary objective is to assess equivalency between total exposure (of tacrolimus area under the curve concentration (AUC0-24)), immediate-release tacrolimus (Prograf^®^) therapy, and prolonged-release tacrolimus (Envarsus^®^) using a daily dose conversion factor of 0.7 for prolonged- versus immediate-release tacrolimus. Secondary objectives are the assessment of pharmacodynamics, pharmacogenetics, adherence, gut microbiome analyses, adverse events (including tacrolimus toxicity and biopsy-proven rejections), biopsy-proven rejections, difference in estimated glomerular filtration rate (eGFR), and occurrence of donor-specific antibodies (DSAs).

**Discussion:**

This study will test the hypothesis that once-daily prolonged-release tacrolimus (Envarsus^®^) is bioequivalent to twice-daily intermediate-release tacrolimus after pediatric kidney transplantation and may reduce toxicity and facilitate medication adherence. This novel concept may optimize immunosuppressive therapy for more stable graft function and increased graft survival by avoiding T-cell mediated and/or antibody-mediated rejection due to improved adherence. In addition, the study will provide data on the pharmacodynamics and pharmacogenetics of prolonged-release tacrolimus in children and adolescents.

**Clinical Trial Registration:**

EUDRA-CT 2019-003710-13 and ClinicalTrial.gov, identifier NCT06057545.

## Introduction

From the first successful kidney transplant in 1954 to the current era of transplantation, there have been tremendous developments in transplant immunology and immunosuppressive therapy. Tacrolimus, a calcineurin inhibitor (CNI), is currently the first-line immunosuppressive agent in kidney transplantation. In children, the start dose of tacrolimus is conventionally determined based on body surface area or weight. Further dosing depends on monitoring of tacrolimus trough levels in blood. Non-adherence to immunosuppressants is associated with the development of T-cell-mediated and/or antibody-mediated rejection after kidney transplantation, which is the major cause of long-term graft loss. The therapeutic index of tacrolimus is narrow. The main goal is to reach/maintain the target blood concentration of tacrolimus without the risk of graft rejection or toxicity. It has already been shown that once-daily, rather than twice-daily drug formulations lead to a significant improvement in adherence (1). Therefore, it is expected that prolonged-release tacrolimus would lead to a more stable tacrolimus exposure and thereby provide a more reliable immunosuppression after kidney transplantation.

The once-daily formulation of tacrolimus (extended-release tacrolimus), Advagraf^®^, was designed to reduce the burden of daily pills compared to twice-daily Prograf^®^. A pediatric study has demonstrated comparable pharmacokinetics when immediate-release tacrolimus was converted to the prolonged-release formulation. The conversion was effective and well tolerated (2). Recently, a new prolonged-release tablet formulation of tacrolimus (Envarsus^®^) using MeltDose™ (US Patent No. 7,217,431), a drug delivery technology that enhances the absorption of fat-soluble drugs, was approved as an immunosuppressive medication after kidney and liver transplantation in adults, but it has not yet obtained approval in children (3). Studies in adults have shown that Envarsus^®^ provides the same therapeutic efficacy as the conventional immediate-release formulation of tacrolimus (Prograf^®^) with improved bioavailability, a more consistent pharmacokinetic profile, and a reduced peak-to-trough profile, which may result in reduced CNI-related toxicity.

In recent years, different protocols combining different immunosuppressive agents have been used in pediatric kidney transplantation with changes over time and large variability between centers and countries (4). The choice of the immunosuppressive regimen is based on efficacy trials as well as personal expertise. Patient stratification to immunosuppressants has rarely been based on the risk of viral infection or metabolic disease but is often tailored to the individual immunologic risk of rejection. To summarize the few randomized pediatric trials on immunosuppression, tacrolimus appears to have advantages over cyclosporine A in pediatric kidney transplantation, steroid-free protocols are feasible in low-immunologic risk, and induction therapy with interleukin-2 (IL-2) receptor antagonists may not be beneficial in a standard-risk population (5). It is therefore important to find the best combination of immunosuppressive agents that optimizes allograft survival by preventing acute rejection while limiting agent toxicities (6).

The inter-individual differences in tacrolimus exposure in pediatric patients are related to patient characteristics, such as weight, age, and laboratory parameters such as hematocrit (Hct), concomitant medications, and pharmacogenetic diversity of the CYP3A4/A5 genes (7). As for intra-patient variability, several factors have been identified, including interactions with concomitant medications and food, diarrhea, and interchangeable administration of generic tacrolimus formulations. There is a wide range of tacrolimus intra-patient variability in adult and pediatric populations from < 5 to > 50%, with average values of 15–30% (7, 8). It needs to be recognized that high tacrolimus intra-patient variability is an important prognostic risk factor after solid organ transplantation (7, 8).

Data on the pharmacokinetics of immediate-release tacrolimus versus prolonged-release tacrolimus in the pediatric population are scarce and not available for immediate-release tacrolimus versus extended-release LCT-tacrolimus. Vondrak et al. showed a 1.3 to 1.9 increase in tacrolimus Tmax for prolonged-release tacrolimus versus immediate-release tacrolimus in pediatric solid organ transplant recipients (9). The main finding of this study was that systemic exposure (AUC0-24) was lower for the early post‐transplant period for the prolonged‐release formulation which equalized by 1 month after transplantation. These findings were consistent with those reported in *de novo* adult transplant recipients (9, 10). In adult transplant recipients, the switch from immediate-release tacrolimus to extended-release LCPT tacrolimus was safe and resulted in a 12.5% to 16% increase in the tacrolimus trough level over total daily dose ratio, indicating improved bioavailability (11, 12). Despite the finding that tacrolimus trough levels remained within the standard reference range for most patients (11), the coefficient of variation for the intra-patient variability did not change significantly after the switch from immediate-release tacrolimus to extended-release LCP tacrolimus (13).

The objective of this study is to evaluate the pharmacokinetic profile, efficacy, and tolerability of Envarsus^®^ in children and adolescents aged ≥ 8 and ≤ 18 years to assess its potential role as an additional option for immunosuppressive therapy after pediatric kidney transplantation.

## Methods/Study design

**Figure 1:**
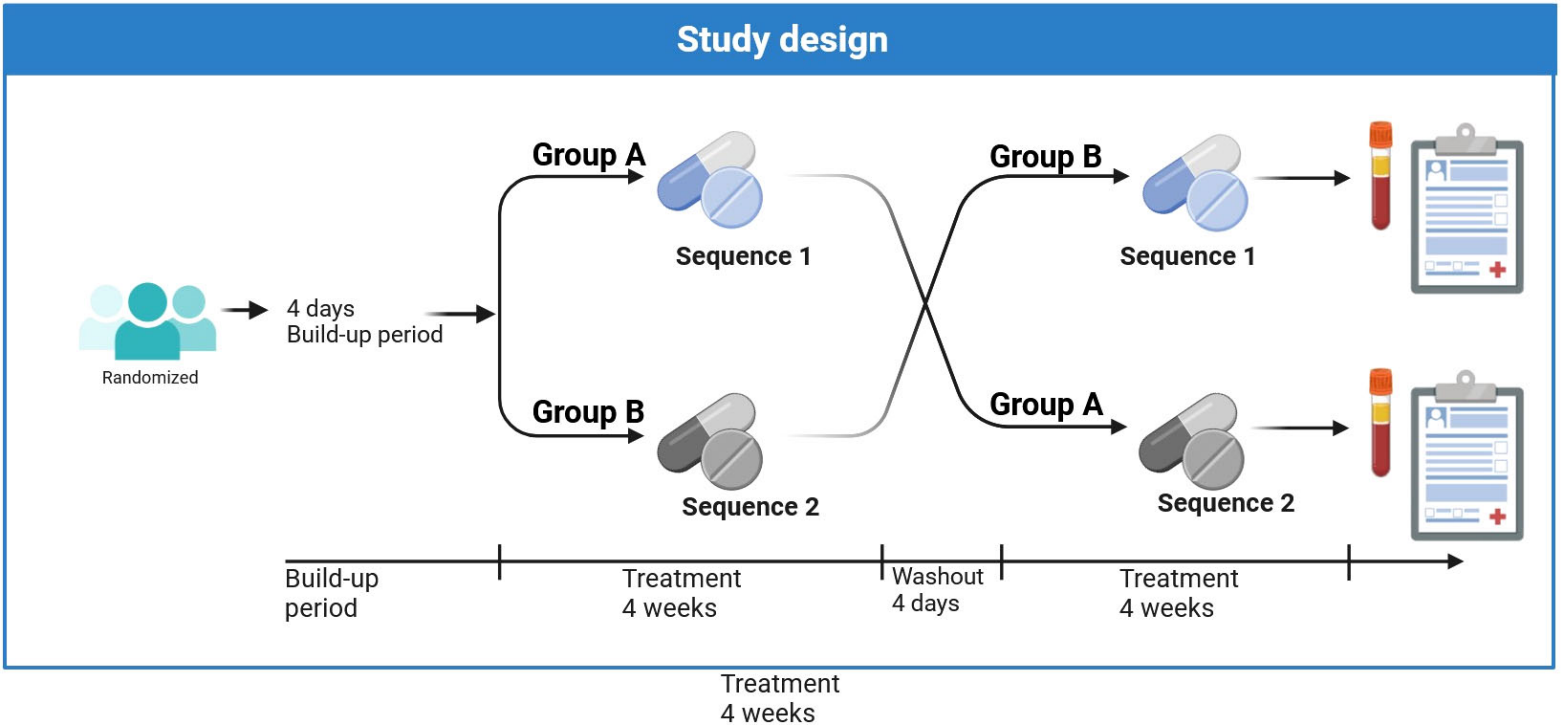
Study design. Sequence 1: 4 weeks Envarsus^®^. Sequence 2: 4 weeks Prograf^®^. The exact timepoints of study visits are given in **Table 2**. At the beginning of the washout period, the medication administered in the first treatment sequence is discontinued and the other medication is started so that a stable build-up is secured.

The study is designed as a randomized, prospective crossover trial. Patients receive two treatment sequences. Sequence 1 includes 4 weeks Envarsus**^®^** and sequence 2 includes 4 weeks Prograf**^®^**. A daily dose conversion factor of 0.7 for prolonged- versus immediate-release tacrolimus is used. Patients are randomized to either group A (sequence 1, followed by sequence 2) or group B (sequence 2, followed by sequence 1) with a wash-out period of 4 days between the two sequences. The trial design is summarized in **Figure 1**. The total study duration per patient (including the build-up periods) is approximately 9 weeks from visit 1. Risks and burdens for study participants have been minimized so that participation in the study over the planned 9-week duration will not exceed routine burdens. The total study duration (first patient first visit: FPFV to last patient last visit: LPLV) will be approximately 20 months, including a recruitment period of approximately 18 months.

The primary objective is to assess whether the tacrolimus area under the curve (AUC0-24) concentration in blood samples collected before and 1.5, 2.0, 4.0, 6.0, 8.0, 12.0, 13.5, 14.0, 16.0, 20.0, and 24.0 hours after administration in compliant patients without any protocol deviations is bioequivalent between twice-daily immediate-release tacrolimus (Prograf^®^) therapy and once-daily prolonged-release tacrolimus (Envarsus^®^) therapy using a daily dose conversion factor of 0.7 for prolonged- versus immediate-release tacrolimus. Envarsus^®^ is available in 0.75, 1.00, and 4.00 mg capsules.

A fast and easy-to-use liquid chromatography-tandem mass spectrometry (LC-MS/MS) method for the determination and quantification of tacrolimus in whole blood was developed and validated. The used method was based on an already published work for the determination of immunosuppressants in serum and plasma (7). Modifications consisted of the use of a different biological matrix (whole blood) with sample preparation and an extraction protocol of commercially available standards and control samples. Sample preparation was based on protein precipitation. A volume of 100µL of whole blood was admixed with 200µL of isotopically labeled internal standard and 200µL zinc sulfate heptahydrate, shaken on a Vortex mixer for 30sec., and pre-dispersed using an external ultrasonic bath for 10min at room temperature, with subsequent centrifugation at 4 000 × g for 15 minutes at 6°C. The analytical performance data of the assay is as follows: Recovery rate: Tacrolimus: 97.9% (2.00 µg/L) and 103% (20.00µg/L); lower limit of quantification (LLOQ in µg/L): 0.56 µg/L tacrolimus; upper limit of quantification (ULOQ in µg/L): 100.0 µg/L tacrolimus. Precision (intra-day-assay): analyte CV in % (at a concentration in µg/L), n=10 per conc.-level: Tacrolimus 3.5 (2.6); 3.4 (7.3); 2.5 (16.7); 1.6 (34.2). Precision (inter-day-assay): analyte CV in % (at a concentration in µg/L), n=10 per conc.-level: Tacrolimus 5.5 (2.6); 3.8 (7.3); 3.7 (16.7);.6 (34.2).

Secondary objectives are to assess Tacrolimus Cmax, efficacy (pharmacodynamics) in terms of residual expression of nuclear factor of activated T-cell (NFAT)-regulated gene expression, potential influence of pharmacogenetics, trough levels and doses of immediate-release tacrolimus (Prograf^®^) compared to prolonged-release tacrolimus (Envarsus^®^), cumulative tacrolimus dose, adherence, adverse events (including tacrolimus toxicity and biopsy-proven rejections), biopsy-proven rejections, differences in estimated glomerular filtration rate (eGFR) (Chronic Kidney Disease in Children (CKiD) formula) from baseline, microbiome analyses, and occurrence of donor-specific antibodies (DSA).

To determine the contribution of genetic factors for interindividual pharmacokinetic variability of tacrolimus exposure observed during the study, genetic variants in *CYP3A5*, *CYP3A4*, and *ABCB1* will be assessed in all children.

### Study drugs

#### Prograf^®^ and Envarsus

Prograf^®^ and Envarsus^®^ contain the active ingredient tacrolimus. Tacrolimus is an immunosuppressive drug that reduces T-cell activity by binding to a cytosolic protein (FKBP12); it specifically and competitively inhibits calcineurin, leading to a calcium-dependent inhibition of T-cell signaling pathways (14, 15).

Prograf^®^, the immediate-release formulation of tacrolimus, exhibits significant inter- and intra-individual variability in absorption due to interactions with food and concomitant medications and the cytochrome P-450 system, which is subject to functional polymorphisms. In addition, Prograf^®^ has rather low bioavailability due to poor water solubility and pre-systemic gastrointestinal metabolism (16, 17). In humans, tacrolimus has been shown to be absorbed throughout the gastrointestinal tract. Available tacrolimus is generally absorbed rapidly.

Envarsus^®^ is a prolonged-release formulation of tacrolimus and was approved by the European Medicines Agency (EMA) in 2014. It is currently indicated for the prophylaxis of transplant rejection in adult kidney or liver allograft recipients and treatment of allograft rejection in adult patients refractory to other immunosuppressive drugs but, to date and in contrast to Prograf^®^, it has not been officially licensed for use in children. The prolonged-release formulation results in an extended oral absorption profile with an average time to maximum blood concentration (C_max_) of approximately 6 hours (t_max_) at a steady state. The oral bioavailability of Envarsus^®^ is approximately 40% higher than the same dose of the immediate-release tacrolimus formulation Prograf^®^ in kidney transplant patients (18). There is a strong correlation between AUC0-24 and steady-state whole blood trough levels for Envarsus^®^. Therefore, monitoring of whole blood trough levels provides a good estimate of systemic exposure (19).

In the systemic circulation, tacrolimus binds strongly to erythrocytes resulting in an approximately 20:1 distribution ratio of whole blood/plasma concentrations. In plasma, tacrolimus is highly bound (>98.8%) to plasma proteins, mainly to serum albumin and α-1-acid glycoprotein (20). The half-life of tacrolimus is comparatively long and variable. In healthy subjects, the mean half-life in whole blood is approximately 30 hours (21). It was found that, following intravenous and oral administration of ^14^C-labeled tacrolimus, most of the radioactivity was excreted in the feces (22).

Tacrolimus has a narrow therapeutic index. Tacrolimus levels below this recommended range carry the risk of graft rejection, while levels above the range are associated with increased toxicity such as opportunistic infections, nephrotoxicity, diabetes, tremor, hypertension, and malignancies (23–25). The improved bioavailability of Envarsus^®^, which allows once-daily dosing, results in lower inter- and intra-patient variability of drug absorption, with reduced peak-to-trough fluctuation and earlier achievement of a stable tacrolimus profile, as demonstrated in healthy volunteers (26). Envarsus^®^ shows an improved pharmacokinetic profile compared to Prograf^®^ with significantly lower peak levels. Therefore, it may be advantageous in terms of toxicity (27).

Regarding the high intra- and inter-patient variability of tacrolimus, increasing clinical evidence suggests that gut bacterial drug metabolism may play a crucial role (28, 29). Experimental results from our group demonstrated tacrolimus metabolization by ubiquitous bacterial species *in vitro* and showed that some strains could even metabolize up to 85% of the parent drug concentration (30). As explained above, the MeltDose™ drug delivery technology of Envarsus^®^ results in higher bioavailability and more consistent pharmacokinetics. This is partly explained by the predominant release in the colon, where the prevalence of CYP3A is significantly lower than in the small intestine (31). As a result, the pre-systemic elimination of Envarsus^®^ due to CYP3A metabolism is significantly reduced. As with the identification of pharmacogenetic factors, the stool microbiome might lead to a potential paradigm shift in the pharmacokinetics of tacrolimus and its respective drug formulations.

### Inclusion/exclusion criteria

A total number of 30 Caucasian male or female children at four German study sites will be enrolled in this clinical trial. They will be enrolled if they meet the inclusion/exclusion criteria listed in **Table 1**. Dynamic randomization according to the method proposed by Signorini will be used to assign patients to the respective arm. The study will be randomized with a probability of 50:50. The total study duration (first patient first visit (FPFV) to last patient last visit (LPLV)) will be approximately 20 months, including a recruitment period of around 18 months. The study duration per patient will be approximately 9 weeks from visit 1.

**Table 1:** Selection criteria.

**Inclusion Criteria**
1. Pediatric kidney transplant recipients (single-organ recipients).
2. Aged ≥ 8 years but ≤ 18 years who are receiving tacrolimus (Prograf^®^) therapy and who are able to swallow tablets with a minimum dose of 0.75 mg/day Envarsus^®^.
3. At least 6 months after transplantation.
4. Stable kidney function (delta eGFR < 10 ml/min/1.73 m^2^ (CKiD formula) over the last 3 months.
5. Women of childbearing potential who are practicing complete abstinence from sexual intercourse (periodic abstinence and withdrawal are not acceptable) or who have a sexual relationship with female partners only and/or with sterile male partners; or women of childbearing potential who are sexually active with a fertile male partner, who have a negative pregnancy test during screening and who agree to use reliable methods of contraception from the time of screening, during the study and for a period of 4 weeks following the last administration of study medication; or women without childbearing potential defined as females before menarche or at least 6 weeks after surgical sterilization by bilateral tubal ligation or bilateral oophorectomy or hysterectomy or uterine agenesis.
6. Patient/parents/legal guardian(s) must be capable of understanding the purpose and risks of the study.
7. Signed informed consent was obtained by the patient and parents/legal guardians.
**Exclusion Criteria**
1. Coefficient of variation of tacrolimus trough levels > 0.35 over the previous 6 months.
2. Pregnancy/breastfeeding.
3. Unstable kidney function.
4. Hypersensitivity to any of the components of the medications used.
5. Not eligible for any reason according to the investigator’s evaluation.
6. Known positive HIV-1 or HCV test.
7. Participation in another clinical trial (other investigational drugs or devices at the time of enrollment or within 30 days prior to enrollment).

### Sample size calculation

The expected ratio of the geometric means of the AUC of Envarsus^®^ vs. Standard tacrolimus is 0.9749. The standard deviation of the coefficient of variation is calculated as 0.303 derived from the corresponding CI (32). The variation coefficient (as the ratio of expected ratio and standard deviation) is then assumed as 0.311. With a two-sided standard error of 10% and the equivalence limits of 0.8 and 1.25 (corresponding to the EMA guideline), a sample size of 27 is calculated to reach a power of 80%. With a dropout of 10%, 30 patients are therefore required.

### Study assessments

Patients will be seen and evaluated according to the flow chart given in **Table 2**. At the time of enrollment, participants are treated with Prograf^®^ to prevent transplant rejection. Patients will be monitored regularly in the outpatient clinic. In this way, potentially eligible patients will be identified during routine outpatient visits.

**Table 2:** Flow chart of study visits.

Phase	0 Screening	1 Baseline	2a Build-up period	2b Sequence 1 (A) or 2 (B) *(hospital stay for AUC0-0-24^6^)*	3a Build-up period	3b Sequence 2 (A) or 1 (B) *(patients hospitalized for AUC0-24^6^)*	4
**Duration**	Within 6 weeks before baseline	1 day (Day 1)	4 days (Day 2-5)	4 weeks /Day 6-33)	4 days (Day 34-37)	4 weeks (Day 38-65)	11 days (Day 66-76)
**Visits**	Screening-Visit	Baseline-Visit	Build-up-Visit 1	AUC0-24 Visit 1 (Hospital stay^6^)	Build-up-Visit 2	AUC0-240 Visit 2 (Hospital stay^6^)	End of study Visit
**Day of visits**		Day 1	Day 4 (+/- 1 day)	Day 19 (+/- 7 days)	Day 36 (+/- 1 day)	Day 51 (+/- 7 days)	Day 71 (+/-5 days)
Informed Consent	✓						
Inclusion/ Exclusion Criteria	✓						
Randomization		✓					
Physical Examination^1a^		✓	✓	✓	✓	✓	✓
Baseline Characteristics^1b^		✓					
Blood Values^2a^		✓	✓	✓	✓	✓	✓
Urine Values^2b^		✓	✓	✓	✓	✓	✓
Urine Pregnancy Test		✓	✓	✓	✓	✓	✓
Urine Volume 24h^2c^		✓		✓		✓	
Full AUC0-240-240-24^2d^ (serial blood samples at 0, 1.5, 2, 4, 6, 8, 12, 13.5, 14, 16, 20, 24 h)				✓		✓	
Pharma-cogenetic Analysis^2e^		✓					
Trough Levels^2f^				✓			
Microbiome Analysis (feces, urine, blood)^2d^		✓		✓		✓	✓
Study Drug Dispensing		✓		✓			
Switch to other Immunosuppres-sant^3^			✓ (Day 2)		✓ (Day 34)		
Concomitant Medication		✓	✓	✓	✓	✓	✓
Envarsus^®^ Return^4^					✓ (for Group A)		✓ (for Group B)
Adverse Events^5^	✓	✓	✓	✓	✓	✓	✓

^1a^Complete physical examination, vital signs (blood pressure, pulse), weight, height, date, and time of visit.

^1b^Medical history including underlying disease, date of transplantation, type of transplantation (living donation/deceased donation), preemptive transplantation (yes/no), gender, date of birth, and clinical assessment.

^2a^Blood values: blood gas analysis, creatinine, urea, LDH, cystatin C, eGFR (CKiD formula), HLA and non-HLA-DSA, ALT, yGT, CK, HbA1c, hematology (incl. differential blood count), electrolyte (potassium, calcium, phosphate, magnesium), CRP, and tacrolimus trough level.

^2b^Urine Values: Urea, glucose, creatinine, and albumin.

^2c^Urine Volume: 24h collection urine.

^2d^Full AUC0-240-240-24: Patients need to be hospitalized for the sampling of blood (9.5 ml whole blood per patient) to determine tacrolimus levels; time = 0 is defined as 12 hours after the last intake of Prograf^©^/24 hours after the last intake of Envarsus^©^. Pharmacokinetic Analysis: 0.5 ml whole blood (EDTA) per sampling point (0.0, 1.5, 2.0, 4.0, 6.0, 8.0, 12.0, 13.5, 14.0, 16.0, 20.0, and 24.0 h) to be stored at -20° C until shipping (to be shipped frozen to Cologne, Labor für Therapeutisches Drug Monitoring. Pharmacodynamic Analysis: 0.5 ml whole blood (lithium-heparin) per sampling point (0.0, 1.5, 2.0, 4.0, 6.0, 20.0, and 24.0 h) to be stored at room temperature and to be shipped later the same day to Heidelberg Institute of Human Genetics.

^2e^Pharmacogenetic Analysis: At baseline visit 1 ml whole blood (EDTA) for pharmacogenetic analysis (to be shipped at room temperature.

^2f^Trough (C_0_) levels: defined as 12 hours after the last intake of Prograf^©^/24 hours after the last intake of Envarsus^©^. 0.5 ml whole blood (EDTA) to be stored at -20° C until shipping (to be shipped frozen).

^2g^Microbiome Analysis: fecal samples: metagenomic-/transcriptomic analysis and metabolomics; urine and blood samples: metabolomics.

^3^In phase 3b, patients will be switched to the other immunosuppressant for 4 weeks.

^4^Patients must return the unused Envarsus^®^ for accountability and destruction of unused tablets at Visit 3a for Group A and also at Visit 4 for Group B.

^5^The adverse event documentation period for this trial begins with informed consent and ends with the last study visit.

^6^Patient hospitalizations must happen from Monday to Wednesday as samples for pharmacodynamic analysis must be shipped immediately within 1 day and must reach the central lab in Heidelberg on Thursday at the latest.

A complete physical examination including vital signs (blood pressure and pulse), weight, height, date, and time of visit will be documented at each visit. Screening evaluations will be performed within 6 weeks prior to the baseline evaluation to determine the patient’s eligibility to participate in the study. At each visit, the following laboratory measurements will be performed at the local laboratory: Cystatin C, eGFR (CKiD formula), hematology (including differential blood count), electrolyte (potassium, calcium, phosphate, and magnesium), human leukocyte antigen (HLA) and non-HLA-DSA, alanine aminotransferase (ALT), gamma-glutamyl transferase (yGT), creatine kinase (CK), hemoglobin A1c (HbA1c), blood gas analysis, creatinine, urea, hematology, electrolytes, lactate dehydrogenase (LDH), C-reactive protein (CRP), and tacrolimus trough level, and in urine: urea, glucose, creatinine, and albumin including pregnancy test. Tacrolimus trough levels (C_0_) will be determined at 12 hours after the last intake of Prograf^®^ or 24 hours after the last intake of Envarsus^®^, respectively. Some special analyses will be carried out in the central laboratory in Cologne (pharmacokinetics; Laboratory for Therapeutic Drug Monitoring, University Hospital of Cologne, Germany) and Heidelberg (pharmacodynamics; Institute of Immunology, University Hospital of Heidelberg, Germany). The pharmacodynamic analysis will be based on the residual expression of NFAT-regulated genes. Gut microbiome analyses will be performed at the European Molecular Biology Laboratory (EMBL) in Heidelberg. Fecal samples will be collected at home 0-48 hours prior to the scheduled visit. Information on concomitant medication use is documented for each patient. Envarsus^®^ (prolonged-release tablets containing tacrolimus (0.75, 1.00, or 4.00 mg)) should be taken orally once daily in the morning on a fasting state at 24-hour intervals. Prograf^®^ (hard capsules containing tacrolimus in commercially available strengths) should be taken orally twice daily at 12-hour intervals, morning and evening, on an empty stomach; dosing according to the Summary of Product Characteristics.

### Statistical methods

The primary objective of the study is to demonstrate the equivalency between total exposure and trough levels of both Prograf^®^ and Envarsus^®^ (with a daily dose conversion factor of 0.7) in compliant patients without any protocol deviations. Therefore, the primary endpoint is the full AUC0-24 of tacrolimus levels over 24 hours. The objective is to demonstrate that the 90% confidence interval for the ratio of the full AUC0-24 of Envarsus^®^ (with a conversion factor of 0.7 for the daily dose) to Prograf^®^ is within the acceptance interval of 0.8 - 1.25 (33). This interval has been chosen in accordance with the German Federal Institute for Drugs and Medical Devices. As a secondary analysis, whether the confidence interval is also within the narrower interval of 0.9 to 1.11 recommended for drugs with a narrow therapeutic index (NTI) will be checked (33).

Demographic and baseline characteristics will be summarized as absolute and relative frequencies (for categorical outcomes), median and interquartile ranges (IQR), or arithmetic means and standard deviation. Pharmacokinetic measures will be summarized as median, geometric mean, arithmetic mean and standard deviation, minimum and maximum, and coefficient of variation. The primary endpoint – full AUC0-24 – will be calculated from tacrolimus measurements after administration of the respective medication by linear interpolation between measurement time points (see **Supplementary Material**). It will then be analyzed using an ANOVA model on the log-transformed AUC0-24 values comparing Prograf^®^ and Envarsus^®^ with sequence, subject within sequence, and period as further fixed factors as suggested by the EMA guideline (33). A summary of the assessment of the primary objective in terms of the estimand framework is given in the **Supplementary Material**.

In addition to full AUC0-24 analysis, the following pharmacokinetic parameters will form part of the evaluation: C_0_, C_max_, t_max_, and their corresponding coefficients of variation. Pharmacokinetic half-life and apparent clearance after oral administration of Envarsus^®^ and Prograf^®^ will be evaluated accordingly. Pearson’s correlation coefficients will be used to assess suitability and trough levels and AUC0-24 will be correlated.

Low NFAT expression indicates high immunosuppression and high NFAT expression indicates low immunosuppression. The tacrolimus effect is therefore complementary to the NFAT transcripts (E=1-NFAT%). The residual NFAT expression at each time point will be compared to the corresponding tacrolimus blood concentration to estimate the peak (E_peak_) and trough (E_trough_) effect. With the help of the sigmoid E_max_ model, the immunosuppressive effect will be estimated as the percentage of E_max_ (E=%E_max_).

A test for univariate associations between changes in the gut microbial abundance profile will be performed during the switch from Prograf^®^ to Envarsus^®^ compared to changes during the pre- and post-switch time intervals (Wilcoxon test). We will also include Prograf^®^ AUC0-24 and *CYP3A* genotypes as additional predictors in these models to quantify whether microbial AUC0-24 predictors are consistent or different between Prograf^®^ and Envarsus^®^ AUC0-24 and the extent to which gut bacterial drug metabolism is independent of host metabolism. We will attempt to identify gut microbial correlates of adverse events and rejection, but the analyses will be exploratory.

All persons performing the analyses will be blinded to the sequence order. The statistical analysis plan, in which all planned analyses are described in detail, is finalized before the database lock.

## Discussion

Non-adherence to immunosuppressive therapy, particularly in adolescents, can lead to the development of T-cell-mediated and/or antibody-mediated rejections after kidney transplantation, which is the major cause of long-term graft loss.

Once-daily prolonged-release formulation Envarsus^®^ has the potential to improve adherence but is not currently approved for use in adolescents under 18 years of age. Pro-Tac, our multi-center interventional trial, will test the hypothesis that once-daily prolonged-release tacrolimus (Envarsus^®^) is equivalent to intermediate-release tacrolimus and may result in improved drug adherence, a more consistent pharmacokinetic profile, and reduced peak-to-trough after kidney transplantation compared to twice-daily immediate-release tacrolimus formulation (Prograf^®^). This novel concept may optimize immunosuppressive therapy for more stable graft function and improved graft survival.

### Ethics and dissemination

This trial is being conducted in accordance with the German Drug Law (AMG), the German Good Clinical Practice (GCP-V), the International Conference on Harmonization (ICH), GCP guidelines, and other applicable ethical and regulatory requirements. A favorable assessment from the International Electrotechnical Commission (IEC) and approval from the National Competent Authority (NCA) were obtained before the start of the trial. The trial has received a positive primary ethics opinion from the Ethics Committee of the University Hospital of Essen (21-10167-AF) and positive secondary ethics judgments from the Ethics Committees of the University Hospitals of Heidelberg, Hamburg, Cologne, and Essen.

Informed consent is obtained from all participating patients and their parents. Monitoring, audits, and inspections are performed for reasons of quality assurance and to verify that the study is conducted according to the protocol as well as to legal and regulatory requirements applicable to clinical trials.

The study is registered under EUDRA-CT 2019-003710-13 and ClinicalTrial.gov Identifier: NCT06057545.

The results of the study are planned to be published in a Pubmed listed open-source publication.

### Trial status

The first patient was enrolled on 25th April 2023. To date (September 2023), four patients have been enrolled in Essen, Cologne, and Heidelberg.

## Ethics statement

The studies involving humans were approved by Ethics committee of the Medical Faculty of the University of Duisburg-Essen. The studies were conducted in accordance with the local legislation and institutional requirements. Written informed consent for participation in this study was provided by the participants’ legal guardians/next of kin.

## Author contributions

SK: Writing – original draft. BT: Writing – original draft. TA: Writing – review & editing. MB: Writing – review & editing. BB: Conceptualization, Writing – review & editing. AB: Writing – review & editing. LE: Writing – review & editing. TG: Writing – original draft. SL: Writing – original draft. CM: Writing – original draft. JO: Writing – review & editing. AZ: Writing – original draft. LW: Writing – original draft. LP: Conceptualization, Supervision, Writing – review & editing.

## Glossary

**Table:**

AMG	German Drug Law
ALT	alanine aminotransferase
AUC0-24	area under the curve
C_0_	trough level
CK	creatine kinase
CKiD	Chronic Kidney Disease in Children
C_max_	maximum blood concentration
CNI	calcineurin inhibitor
CRP	C-reactive protein
DSA	donor-specific antibodies
EMBL	European Molecular Biology Laboratory
e.g.	exempli gratia
eGFR	estimated glomerular filtration rate
EMA	European Medicines Agency
E_peak_	estimate the peak of the blood concentration
E_trough_	estimate trough level
FPFV	first patient first visit
GCP	Good Clinical Practice
yGT	gamma-glutamyl transferase
h	hours
HbA1c	hemoglobin A1c
HLA	human leukocyte antigen
ICH	International Conference on Harmonisation
IEC	International Electrotechnical Commission
IL-2	interleukin 2
IQR	interquartile range
LDH	lactate dehydrogenase
LPLV	Last patient last visit
NCA	National Competent Authority
NFAT	nuclear factor of activated T-cells
t_max_	time to peak drug concentration
