# Supplementary material for: A multi-center interventional study to assess pharmacokinetics, effectiveness, and tolerability of prolonged-release tacrolimus after pediatric kidney transplantation: study protocol for a prospective, open-label, randomized, two-phase, two-sequence, single dose, crossover, phase III b trial
Source: Front Nephrol. 2024 Feb 20;4:1331510. doi: 10.3389/fneph.2024.1331510 (PMC10912931; doi:10.3389/fneph.2024.1331510)
Supplement: Supplementary file 1 [file DataSheet_1.docx]

**Primary Estimand:**

The primary clinical question of interest is: Are prolonged-release tacrolimus (Envarsus^®^) treatment (conversion factor of daily dose 0.7) and immediate-release tacrolimus (Prograf^®^) bioequivalent in caucasian paediatric kidney transplant recipients?

The estimand is described by the following attributes:

Population:

Caucasian paediatric kidney transplant recipients under tacrolimus therapy with the following in- and exclusion criteria:

Inclusion Criteria

1. Paediatric kidney transplant recipients (single-organ recipients).
2. Aged ≥ 8 years but ≤ 18 years who are under tacrolimus (Prograf®) therapy and who are able to swallow tablets with a minimum dose of 0.75 mg / day Envarsus®.
3. At least 6 months after transplantation.
4. Stable kidney function (delta eGFR < 10 ml/min/1.73 m2 (CKID formula) over the last 3 months.
5. Women of childbearing potential who is practicing true abstinence from sexual intercourse (periodic abstinence and withdrawal are not acceptable) or who has sexual relationship with female partners only and/or with sterile male partners; or women of childbearing potential and sexually active with fertile male partner who have a negative pregnancy test during screening and who agree to use reliable methods of contraception from the time of screening, during the study and for a period of four weeks following the last administration of study medication; or women without childbearing potential defined as females before menarche or at least 6 weeks after surgical sterilization by bilateral tubal ligation or bilateral oophorectomy or hysterectomy or uterine agenesis.
6. Patient/parents/legal guardian(s)1 must be capable of understanding purpose and risks of the study.
7. Signed informed consent obtained by patient and parents/legal guardians.

Exclusion criteria

1. Coefficient of variation of tacrolimus trough levels > 0.35 over the previous 6 months.
2. Pregnancy/breast feeding.
3. Instable kidney function.
4. Hypersensitivity to any of the components of the medications used.
5. Not eligible for any reason according to the investigator’s valuation.
6. Known positive HIV-1 or HCV test.
7. Participation in another clinical trial (other investigational drugs or devices at the time of enrolment or within 30 days prior to enrolment).

Treatment condition:

The steady state conditions of treatment with Envarsus® (prolonged-release tacrolimus) and treatment with Prograf® (immediate-release tacrolimus) are compared within the same patients.

Variable:

Full tacrolimus AUC calculated from Tac measures before administration of drug and 1.5, 2, 4, 6, 8, 12, 13.5, 14, 16, 20, 24 hours after administration of drug at the time point of 2 weeks (14 ± 7 days) after end of build-up period for each patient under both treatments within two time periods with each a length of 4 weeks

Summary measure:

The summary measure on population level is the 90 % confidence interval of the ratio of the geometric means of the full tacrolimus AUC under Envarsus® treatment (conversion factor 0.7) and the full tacrolimus AUC under Prograf® treatment resulting from the ANOVA model on log transformed AUC values, with group (A and B), subject within group, and sequence (1 and 2) as further fixed effects. It should lie within the boundaries 0.8 to 1.25.

Intercurrent events and strategies:

o Drop-out (see definitions in section 6.4): principle stratum strategy

o Evaluable data only for one sequence: principle stratum strategy

o Any dose adjustment of study medication in either sequence: principle stratum strategy

o Non-compliance in usage (intake of less than 90% of IMP): principle stratum strategy

o Missing of single isolated values of tacrolimus blood concentration measures: hypothectical strategy (linear interpolation from neighbouring values)

Rationale for estimand:

Interest lies in comparability of bioavailability of prolonged-release tacrolimus (Envarusus®) and immediate-release tacrolimus (Prograf®) using a conversion factor of daily dose of 0.7. Therefore, non-adherent participants, participants with dose adjustments or completely missing data on either sequence or Drop-outs will not be included in the primary analysis (principle stratum strategy). If only single values for the determination of the AUC are missing, interpolation is regarded as appropriate method for imputation of hypothetical values. This hypothetical strategy is reasonable since this is the method for AUC calculation anyway and the measurement intervals are narrow.

Summary

The main underlying question can be formulated as follows:

In caucasian paediatric kidney transplant recipients under tacrolimus therapy, does the 90% confidence interval of the ratio of geometric means of the full tacrolimus AUC calculated from Tac measures before administration of drug and 1.5, 2, 4, 6, 8, 12, 13.5, 14, 16, 20, 24 hours after administration of drug at the time point of 2 weeks (14±7 days) after end of build-up period under Envarsus® treatment (conversion factor 0.7) and the full tacrolimus AUC calculated from Tac measures before administration of drug and 1.5, 2, 4, 6, 8, 12, 13.5, 14, 16, 20, 24 hours after administration of drug at a time point of 2 weeks (14±7 days) after end of build-up period under Prograf® treatment lie within the boundaries 0.8 to 1.25 by accounting for treatment sequence (i.e. each patient gets both treatments within two time periods with a length of 4 weeks) while excluding patients with:

• Occurrence of AEs (adverse events) or SAEs (serious adverse events) which preclude study treatment or further study participation

• Occurrence of any of the predefined exclusion criteria

• Acute humoral or cellular rejection episodes that require change of immunosuppressive therapy

• Infections that require change of immunosuppressive therapy

• Discontinuation of tacrolimus

• Use of concomitant medication that interacts with tacrolimus

• Graft loss / begin of dialysis

• Evaluable data only for one sequence

• Occurrence of dose adjustment in either sequence

• Non-compliance in usage (intake of less than 90% of IMP)
